# Supplementary material for: Forecasting off-target drug toxicity using proteomic and genetic data: insights from Torcetrapib
Source: medRxiv. 2025 Dec 4:2025.12.03.25341213. Preprint. [Version 1] doi: 10.64898/2025.12.03.25341213 (PMC12704646; doi:10.64898/2025.12.03.25341213)
Supplement: 1 [file NIHPP2025.12.03.25341213V1-supplement-1.pdf]

medRxiv preprint doi: https://doi.org/10.64898/2025.12.03.25341213; this version posted December 4, 2025. The copyright holder for this preprint (which was not certified by peer review) is the author/funder, who has granted medRxiv a license to display the preprint in perpetuity.
It is made available under a CC-BY 4.0 International license .

Supplemental Figure 1: MR of lipid levels on proteins identified 70 proteins of the 200 differentially expressed proteins were causally impacted by lipid levels.

[High Quality Image]

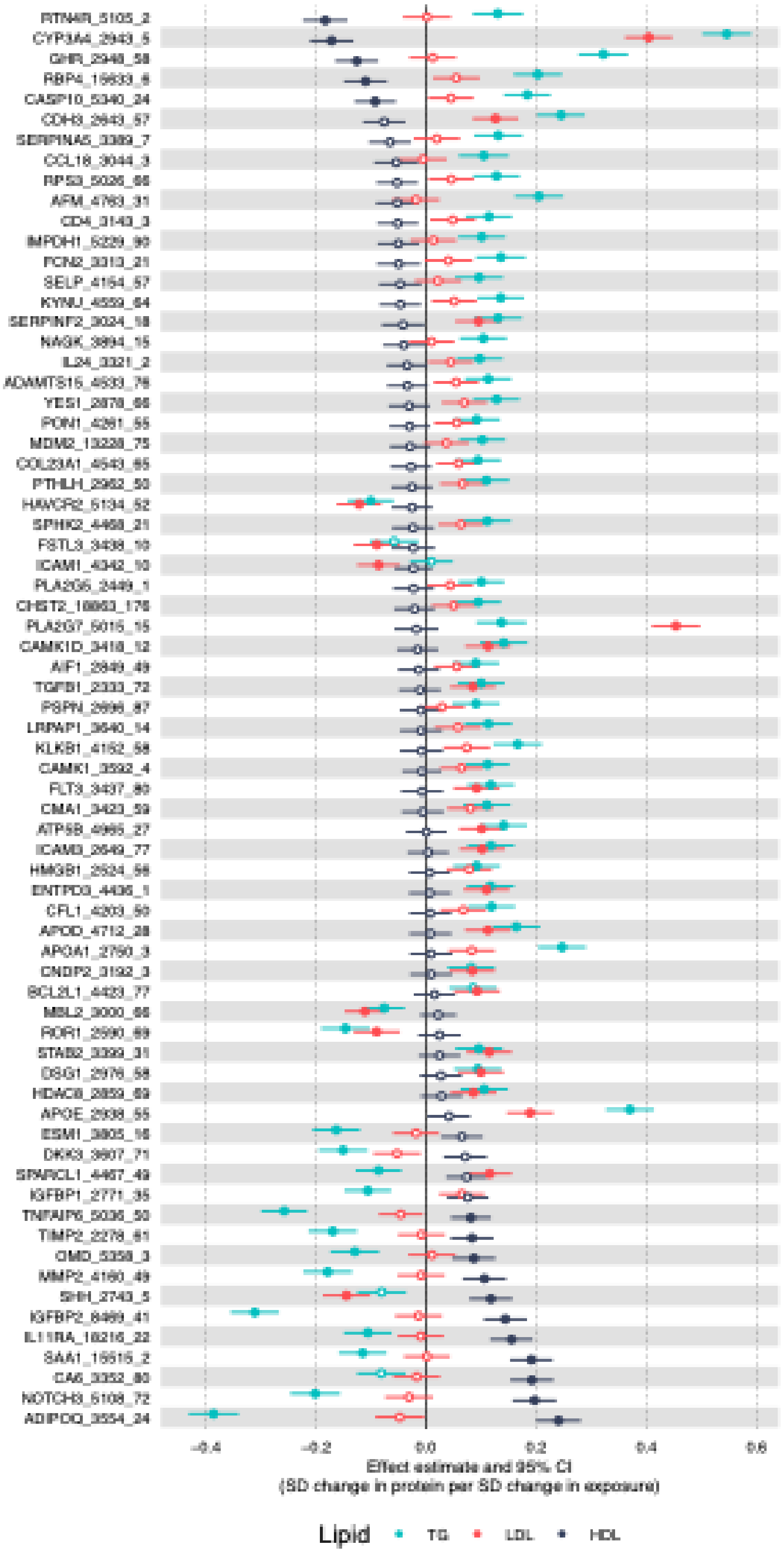

medRxiv preprint doi: https://doi.org/10.64898/2025.12.03.25341213; this version posted December 4, 2025. The copyright holder for this preprint (which was not certified by peer review) is the author/funder, who has granted medRxiv a license to display the preprint in perpetuity.
It is made available under a CC-BY 4.0 International license .

Forest plot showing Mendelian randomization estimates for the causal effects of genetically predicted lipid levels (LDL cholesterol, HDL cholesterol, and triglycerides) on protein levels. Effect sizes represent the change in protein levels (in standard deviations) per standard deviation increase in each lipid fraction. Seventy proteins showed significant associations with at least one lipid fraction after Bonferroni correction for three lipids and 196 tested proteins. Proteins are ordered by ascending HDL cholesterol effect size. Points represent effect estimates with 95% confidence intervals: filled points indicate significant associations, and open points indicate non-significant associations.
